# The Medical Students’ Vade Mecum, or Manual of Examinations, &c., &c.

**Published:** 1847-08

**Authors:** 


ARTICLE XI.

*The Medical Students' Vade Mecum, or Manual of Examinations upon Anatomy, Physiology, Chemistry, Materia Medica, Surgery, Obstetrics, Practice of Medicine, (including Physical Diagnosis and Diseases of the Skin,) and Poisons.* Second Edition, Revised and Enlarged. By George Mendenhall, M.D., Lecturer on Pathology in the Medical Institute of Cincinnati, Member of the Philadelphia Medical Society, &c., &c. Philadelphia: Lindsay & Blakiston. 1847. pp. 574. (From the Publishers.)

The Medical Students' Vade Mecum is a work of very nearly the same character as the Hand Book of Anatomy. In the language of the author, "it is intended to furnish the student of medicine with a short and succinct view of the most important facts and principles which engage his attention during his pupilage, in order that he may refresh and fix more firmly upon his memory what he has read and heard." We unhesitatingly say, that we consider the work well adapted for the purpose for which it is intended. H.

